# Combining Antigen-Based Therapy with GABA Treatment Synergistically Prolongs Survival of Transplanted ß-Cells in Diabetic NOD Mice

**DOI:** 10.1371/journal.pone.0025337

**Published:** 2011-09-22

**Authors:** Jide Tian, Hoa Dang, Daniel L. Kaufman

**Affiliations:** Department of Molecular and Medical Pharmacology, University of California Los Angeles, Los Angeles, California, United States of America; University of London, St George's, United Kingdom

## Abstract

Antigen-based therapies (ABTs) very effectively prevent the development of type 1 diabetes (T1D) when given to young nonobese diabetic (NOD) mice, however, they have little or no ability to reverse hyperglycemia in newly diabetic NOD mice. More importantly, ABTs have not yet demonstrated an ability to effectively preserve residual ß-cells in individuals newly diagnosed with type 1 diabetes (T1D). Accordingly, there is great interest in identifying new treatments that can be combined with ABTs to safely protect ß-cells in diabetic animals. The activation of γ-aminobutyric acid (GABA) receptors (GABA-Rs) on immune cells has been shown to prevent T1D, experimental autoimmune encephalomyelitis (EAE) and rheumatoid arthritis in mouse models. Based on GABA's ability to inhibit different autoimmune diseases and its safety profile, we tested whether the combination of ABT with GABA treatment could prolong the survival of transplanted ß-cells in newly diabetic NOD mice. Newly diabetic NOD mice were untreated, or given GAD/alum (20 or 100 µg) and placed on plain drinking water, or water containing GABA (2 or 6 mg/ml). Twenty-eight days later, they received syngenic pancreas grafts and were monitored for the recurrence of hyperglycemia. Hyperglycemia reoccurred in the recipients given plain water, GAD monotherapy, GABA monotherapy, GAD (20 µg)+GABA (2 mg/ml), GAD (20 µg)+GABA (6 mg/ml) and GAD (100 µg)+GABA (6 mg/ml) about 1, 2-3, 3, 2-3, 3-8 and 10-11 weeks post-transplantation, respectively. Thus, combined GABA and ABT treatment had a synergistic effect in a dose-dependent fashion. These findings suggest that co-treatment with GABA (or other GABA-R agonists) may provide a new strategy to safely enhance the efficacy of other therapeutics designed to prevent or reverse T1D, as well as other T cell-mediated autoimmune diseases.

## Introduction

Antigen-based therapies for autoimmune disease are theoretically appealing because they can induce regulatory responses with little adverse side-effects. When given to young NOD mice ABTs can effectively prevent the development of T1D. ABTs, however, have little or no ability to slow disease progression after NOD mice develop mild hyperglycemia. In human clinical trials, ABT's have not yet shown a significant ability to preserve residual insulin-producing ß-cells in individuals newly diagnosed with T1D. In light of these results, it is widely thought that the most promising future therapeutic approach to preserve residual ß-cells after T1D onset will be through a combination of treatments that induce different beneficial mechanisms [1].

We have shown that T cells express GABA_A_ receptors (GABA_A_-Rs) that can be modulated pharmacologically in the same way as GABA_A_ receptors on neurons [2]. We demonstrated that GABA administration after the onset of insulitis effectively inhibited the development of T1D long-term in NOD mice [3]. Additionally, a GABA_A_-R agonist that can pass through the blood brain barrier ameliorated EAE after its onset in SJL mice [4]. Recently, we showed that GABA consumption through drinking water effectively reduced the frequency and severity of collagen-induced arthritis in DBA1/J mice [5], and that GABA consumption can inhibit the development of obesity-related inflammation [6]. Thus, GABA treatment can inhibit pathogenic immune responses in different animal models of autoimmune disease that are mediated by different mechanisms, which occur in mice with different genetic backgrounds. This, together with GABA's safety for human consumption, makes GABA an excellent candidate for testing in combination with ABTs.

NOD mice often progress from mild to severe hyperglycemia within 1-2 weeks, but it takes about 10–14 days for ABTs to induce maximal immune responses to the administered autoantigen, and even longer for regulatory T cell responses to then spread to other ß-cell autoantigens (reviewed in [7]). Consequently, by the time ABT-induced regulatory responses peak in NOD mice, insufficient ß-cell mass remains and the treatments appear ineffective. However, we, and others, have shown that ABTs using GAD can induce regulatory responses after T1D onset in NOD mice, as evidenced by their ability to prolong the survival of syngenic islet grafts in NOD mice [8], [9]. In these studies, diabetic NOD mice were treated with GAD, maintained on insulin, and several weeks later, the mice were implanted with syngenic islets, thereby allowing sufficient time for the induction and spreading of regulatory responses. Thus, ABT can induce regulatory responses in diabetic NOD mice and these responses can be assessed using a syngenic islet transplantation model. We therefore used this model to assess whether combining ABT with GABA treatment could better protect transplanted ß-cells in diabetic animals than each monotherapy.

## Materials and Methods

All studies were approved by the UCLA Chancellor's Animal Research Committee (approval #2000-023-33). Human GAD65 (Diamyd Medical, Stockholm) was complexed with alum (Pierce Biotechnology, Rockford, Ill.) as per manufacturer's instructions. Female NOD mice (Taconic Farms) were maintained in a specific pathogen-free facility. After the NOD mice developed hyperglycemia (blood glucose >300 mgs/dL on two consecutive days) they were randomly assigned to a treatment group. Mice were, or were not, immunized intraperitoneally with GAD/alum (20 mg or 100 mg) and given plain drinking water or water containing GABA (2 or 6 mg/ml, Sigma-Aldrich catalog #A2129). Fresh drinking water was provided weekly. After 14 days, the mice were revaccinated with the same dose of GAD/alum. Mice were given a minimal amount of insulin (<1.5 units/day) to maintain their blood glucose levels <400 mg/dL. Twenty–eight days after the first treatment, we transplanted 12–13 pancreases from newborn NOD.scid mice under their kidney capsule and insulin treatment was discontinued. Their blood glucose was monitored 3 times per week. Two readings >250 mgs/ml of glucose was considered as the recurrence of hyperglycemia. The extent to which each treatment restored normoglycemia was analyzed by the log rank test. A p<0.05 was considered statistically significant.

## Results

**Figure 1:**
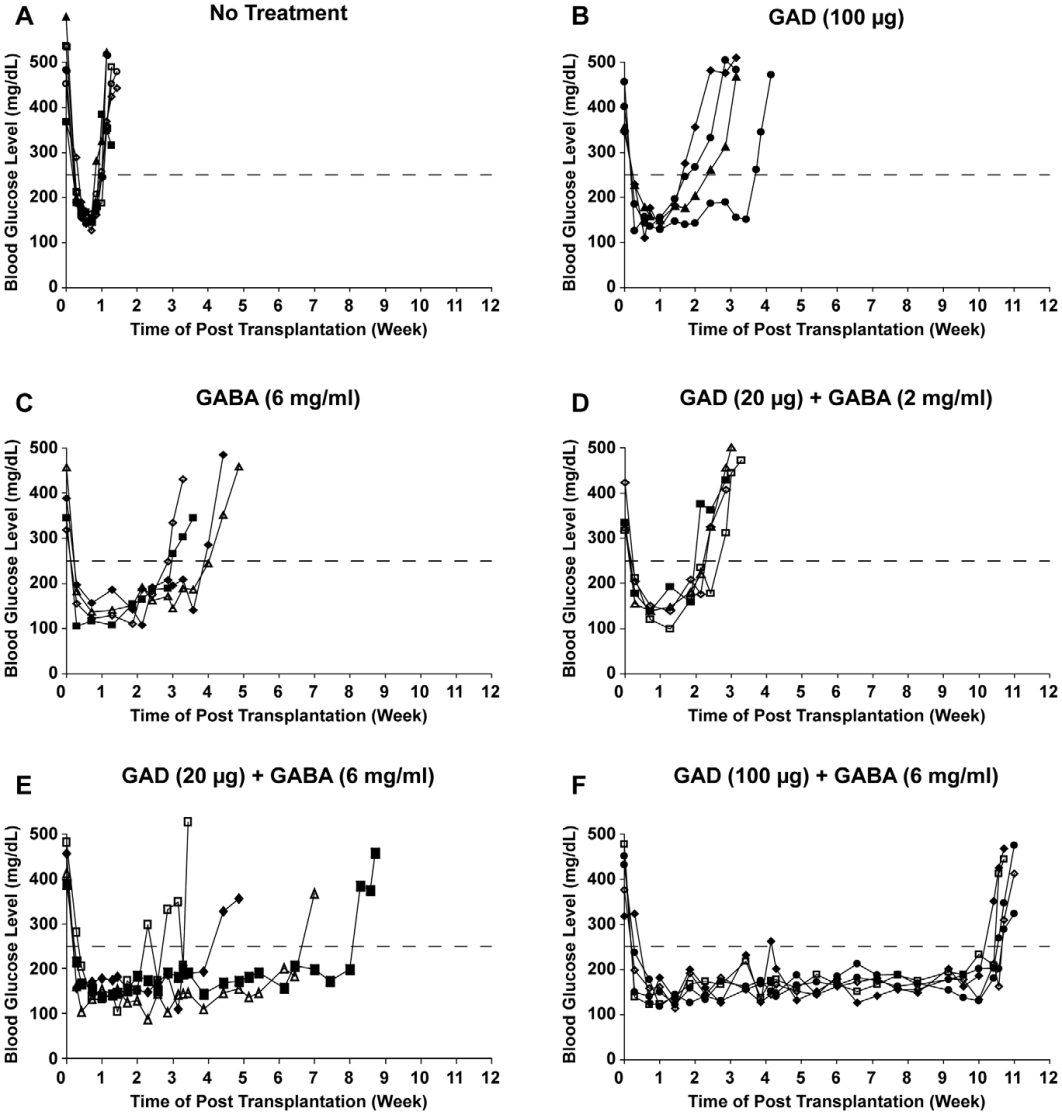
Synergistic effects of combined GAD/alum+GABA treatment to prolong transplanted syngenic ß-cell survival in diabetic NOD mice. After the onset of hyperglycemia mice were, or were not, immunized with GAD/alum (20 or 100 µg, twice) and given drinking water that contained 0, 2 or 6 mg/ml GABA. Mice were maintained on a low amount of insulin until islet transplantation. Twenty-eight days after initiating treatment, the mice received newborn NOD.scid pancreases under their kidney capsule and insulin treatment was discontinued. Lines show longitudinal blood glucose levels of individual mice post-transplantation. Dashed line indicates blood glucose of 250 mgs/dL. A) No treatment, B) GAD/alum (100 µg) alone, C) GABA (6 mg/ml) alone, D) GAD/alum (20 µg)+GABA (2 mg/ml), E) GAD/alum (20 µg)+GABA (6 mg/ml), and F) GAD/alum (100 µg)+GABA (6 mg/ml).

The dynamic changes in the concentration of blood glucose in individual mice within each treatment group after transplantation are shown in Fig. 1. The newborn pancreas grafts reversed hyperglycemia in 100% of the recipient mice. Control transplant recipients became diabetic again about 1 week after receiving the graft, as expected. Mice given GAD/alum (100 µg) monotherapy became diabetic again about 2-3 weeks after transplantation (Fig. 1A) which is a significantly longer period of normoglycemia than that in the control group (p<0.005). Similarly, mice given GABA (6 mg/ml) alone become diabetic again about 3 weeks after transplantation (Fig. 1B, p<0.005 compared to control group). Thus, both GABA and GAD monotherapies can prolong normoglycemia after syngenic pancreas implantation in the absence of immunosuppressants.

Mice that received a combination of low dose GAD/alum (20 µg) with low dose GABA (2 mg/ml) became hyperglycemic 2–3 weeks after receiving the grafts (Fig. 1D, p<0.005 compared to untreated). Although this combined therapy prolonged normoglycemia compared to that in mice that only received a graft, this therapy had similar efficacy to GAD/alum (100 µg) and GABA (6 mg/ml) monotherapies. Interestingly, mice that received low dose GAD/alum (20 µg) together with a higher GABA dose (6 mg/ml) became diabetic sporadically 3-8 weeks post-implantation (Fig. 1E, p<0.01 vs. low dose GAD/alum+low dose GABA). All mice given GAD/alum (100 µg)+GABA (6 mgs/ml) remained normoglycemic for 10-11 weeks post-transplantation (Fig. 1F, p<0.005 vs. GAD/alum (20 µg)+GABA (6 mg/ml). Therefore, while GAD (100 µg) monotherapy and GABA monotherapy (6 mgs/ml) each had a modest but significant beneficial effect, the combination of these treatments acted synergistically to promote longer normoglycemia.

## Discussion

While ABTs are a theoretically appealing strategy to promote ß-cell tolerance, they have not yet demonstrated the ability to preserve residual ß-cells in newly diabetic individuals. Accordingly, there is great interest in combining ABT with other safe treatments in order to more effectively down-regulate pathogenic autoimmune responses. Based on the ability of GABA treatment to inhibit autoimmune processes in mouse models of T1D, EAE and rheumatoid arthritis, and its safety for human consumption, we tested it in combination with an ABT for their ability to protect transplanted ß-cells from autoimmune-mediated rejection in diabetic mice.

We found that monotherapy using GABA or GAD/alum significantly prolonged the survival of transplanted syngenic ß-cells in diabetic NOD mice. While mice that received implants alone remained normoglycemic only about 1 week after transplantation, mice treated with either monotherapy remained euglycemic for about 3 weeks following islet transplantation. Its worth pointing out that while oral GABA monotherapy at 6 mgs/ml had a modest ability to delay the recurrence of hyperglycemia, oral GABA at just 2 mgs/ml was sufficent to very effectively inhibit disease development in the collagen-induced arthritis mouse model [5]. Second, the therapeutic efficacy of GAD (100 µg) in alum was less than that of GAD (100 µg) in incomplete Freund's adjuvant (IFA) which we tested in a previous study using the same syngenic islet transplantation protocol in newly diabetic NOD mice [8]. Immunization with antigen in both alum and IFA leads to vigorous humoral responses to the antigen in mice, but IFA induces five-fold greater Th2 cell responses than alum to the same antigen [10]. Notably, the magnitude of the ABT-induced Th2 response is directly related to their therapeutic efficacy in NOD mice [7], [11], which is likely to explain the greater efficacy of GAD/IFA compared to GAD/alum. Moreover, the magnitude of the ABT-induced Th2 response determines the extent of Th2 spreading to other ß-cell autoantigens which generates additional regulatory responses that can far outnumber those to the administered autoantigen itself [7], [11]. These observations suggest that optimizing adjuvants for ABTs may be critical for inducing effective regulatory responses in human clinical trials.

Combination therapy with a low dose of GAD/alum (20 µg) and GABA (2 mg/ml) had a beneficial effect relative to controls, but it's effect was similar to that of a higher dose of either monotherapy. In contrast, all mice treated with combined GAD/alum (100 µg) and GABA (6 mg/ml) remained euglycemic for 10-11 weeks, suggesting a dose-dependent effect. The period of normoglycemia in graft recipients given this combined therapy was about three-fold longer than in graft recipients given either monotherapy, indicating that ABT and GABA have synergistic beneficial effects.

Combined therapy did not permanently reverse hyperglycemia. However, the therapeutic efficacy compares well with a previous study of combination EGF and gastrin that also used a model of syngenic islet transplantation in diabetic NOD mice and observed that the median time to recurrence of hyperglycemia was about 8 weeks [12]. Pathogenic autoimmune responses in individuals newly diagnosed with T1D, and particularly those with latent autoimmune diabetes in adults (LADA), may be much less aggressive than those in diabetic mouse model that we have studied. Since ABTs and GABA treatments do not induce leukopenia and have no known side-effects, the combination of GABA or other GABA-R agonists with various ABTs are excellent candidates for testing in human clinical trials.

The results indicate that treatment with 100 µg, but not 20 µg, GAD/alum induces significant beneficial effects in diabetic NOD mice (Fig. 1F vs. 1E). Based on a very small dosing study of immunization with GAD/alum to preserve insulin production in individuals with LADA [13], a 20 µg GAD/alum dose was selected for further study in newly diabetic individuals. While this treatment showed initial promise in preserving residual ß-cells in those newly diagnosed with T1D in phase II studies [14], a larger phase III study did not observe a therapeutic effect [15]. Our observations concerning the lack of efficacy of low-dose GAD in alum underscore the need for more basic studies of ß-cell antigen dose and adjuvants in humans to help translate ABTs to the clinic.

Islet cells also express GABA-Rs [16], [17], [18], [19], [20]. *In vitro* studies with isolated islets suggested that GABA could promote islet cell replication [19]. More recent studies indicate that GABA can indeed promote ß-cell replication ([20] and our unpublished observations). However, we do not think that GABA's beneficial effects are primarily through promoting ß-cell replication or modulating islet cell function (e.g., inhibiting glucagon secretion) because 1) transplanted ß-cells are very rapidly destroyed if autoimmune responses are not inhibited (Fig. 1A) and 2) treatment with GABA alone has only a small beneficial effect (Fig. 1C). Accordingly, we believe that GABA's major beneficial effects in this model are through modulating immune cell function.

The protective mechanisms induced by ABTs have been widely studied and can involve the induction of regulatory T cell responses such as Th2, Th3, Tr1 and Tregs (reviewed in [7], [21]). GABA has been shown to inhibit TCR-activated T cell cycling [3]. The opening of T cell GABA_A_-R Cl^-^ channels leads to depolarization, which should modulate the influx of Ca^2+^ through mitogen-activated Ca^2+^ channels and thereby inhibit TCR-activated T cell cycling [3], [22], [23]. In our studies of collagen-induced arthritis in mice we observed that GABA treatment reduced collagen-reactive Th1 responses and reduced collagen-reactive IgG2a (but not IgG1 antibodies, consistent with reduced Th1 help) [5]. Additionally, the activation of GABA_A_-Rs on APC leads to decreased phosphorylation of p38 MAPK and p44/42 ERKs and reduced production of inflammatory cytokines *in vitro* in response to LPS [4]. Finally, we, and others, have recently shown that GABA treatment increases the frequency of Tregs *in vivo* [6], [20]. Since one or more of the GABA treatment-induced protective mechanisms are distinct from the protective mechanisms evoked by other T1D immunotherapeutics that are currently under study, GABA or other GABA-R agonists, are excellent candidates for use in combined therapies to achieve synergistic beneficial effects.

In regards to the safety of GABA treatment, we have observed in past studies that long-term GABA treatment did not significantly alter the total numbers of splenic mononuclear cells and percentages of CD4^+^, CD8^+^, T and B lymphocytes [3], nor did it desensitize autoreactive T cells to GABA-mediated inhibition [5]. Thus, unlike immunodepletive treatments, GABA administration does not reduce the naïve T cell pool that is needed for ABT to prime antigen-specific regulatory T cells. Long-term oral GABA treatment was tested in the 1950s–1980s in human clinical trials for its ability to reduce epileptic seizures and ameliorate cerebrovascular disorders in hundreds of individuals [24], [25], [26]. The results showed no adverse effects, but no clinical benefit, which may because GABA has little ability to cross the blood brain barrier. GABA has been sold for many years over the counter as a nutraceutical with the unsupported claim that it acts as a sleep aide.

Pharmaceutical interests have focused on drugs that can get through the blood brain barrier and modulate GABA-Rs in the central nervous system. The inability of GABA to get through the blood brain barrier makes it an ideal candidate to modulate peripheral GABA-Rs without CNS side effects. Additionally, identifying other GABA-R ligands that do not efficiently cross the blood brain barrier and have better pharmokenetic features may be of value for the treatment of autoimmune and other inflammatory diseases.

Human immune cells express GABA_A_-Rs [27] and GABA inhibits human PBMC inflammatory responses *in vitro* (our unpublished observations). The combined ABT and GABA therapeutic strategy should be extendable to other ß-cell antigens (e.g., proinsulin and HSP277) and to other modes of antigen delivery (e.g., inhalation and DNA). GABA or other GABA-R agonists may also enhance the beneficial effects of other therapeutic approaches. Finally, the therapeutic strategy presented here can be extended to other T cell-mediated autoimmune diseases.
